# Selenocystine against methyl mercury cytotoxicity in HepG2 cells

**DOI:** 10.1038/s41598-017-00231-7

**Published:** 2017-03-10

**Authors:** Han Wang, Beibei Chen, Man He, Xiaoxiao Yu, Bin Hu

**Affiliations:** Key Laboratory of Analytical Chemistry for Biology and Medicine (Ministry of Education), Department of Chemistry, Wuhan University, Wuhan, Hubei 430072 P. R. China

## Abstract

Methyl mercury (MeHg) is a highly toxic substance and the effect of selenium against MeHg toxicity is a hot topic. Until now, no related works have been reported from the view of the point of elemental speciation which is promising to study the mechanism at the molecular level. In this work, to reveal the effect of selenocystine (SeCys_2_) against MeHg cytotoxicity in HepG2 cells, a comprehensive analytical platform for speciation study of mercury and selenium in MeHg incubated or MeHg and SeCys_2_ co-incubated HepG2 cells was developed by integrating liquid chromatography (LC) - inductively coupled plasma mass spectrometry (ICP-MS) hyphenated techniques and chip-based pretreatment method. Interesting phenomenon was found that the co-incubation of MeHg with SeCys_2_ promoted the uptake of MeHg in HepG2 cells, but reduced the cytotoxicity of MeHg. Results obtained by ICP-MS based hyphenated techniques revealed a possible pathway for the incorporation and excretion of mercury species with the coexistence of SeCys_2_. The formation of MeHg and SeCys_2_ aggregation promotes the uptake of MeHg; majority of MeHg transforms into small molecular complexes (MeHg-glutathione (GSH) and MeHg-cysteine (Cys)) in HepG2 cells; and MeHg-GSH is the elimination species which results in reducing the cytotoxicity of MeHg.

## Introduction

Mercury (Hg) is an extremely toxic metal and has been widely used for centuries. People exposure to Hg is mainly due to environmental pollution and the consumption of fish or other aquatic product^1–3^. Methyl mercury (MeHg) is one of the most toxic species of Hg which is assimilated into the water and food, and biomagnifies upwards in food chains^4^. MeHg can induce changes in mitochondria dyshomeostasis, increase the generation of reactive oxygen species, disturb oxidative phosphorylation and electron transport^5, 6^ which lead to lipid peroxidation and cell death^6–8^. It has long been observed that selenium (Se) protects organism from the toxicity of MeHg^9, 10^. The interaction studies involve several Se species such as Se(IV), Se(VI), selenocystine (SeCys_2_), selenomethionine (SeMet) and methyl-Se-cysteine (MeSeCys). The protective effect of Se associates with its presence as a cofactor in enzymes such as glutathione peroxidase and thioredoxin reductase^11–13^ which could protect cellular components from oxidative damage. It is also reported that seleno-compounds could counteract MeHg toxicity by varying the distribution, deposition and excretion of Hg^14–18^.

Animal and cell models are widely used for the study of Hg-Se interactions. Compared with animal models, *in vitro* studies are easier to operate and control, which is beneficial for mechanism study^19^. Cordero-Herrera *et al*.^20^ applied HepG2 cells as model, studied the action of SeMet, SeCys_2_ and MeSeCys against MeHg-induced toxicity. Several biological parameters such as cell viability, lactate dehydrogenase, caspase-3 activity were evaluated and it was shown that SeCys_2_ exerts a protective effect in HepG2 cells against MeHg-induced cell damage. Bulato *et al*.^21^ used LNCaP cells as model, addressed the effect of inorganic Hg on Se utilization in cells supplemented with different Se species (SeMet, MeSeCys and Se(IV)). Kaur *et al*.^22^ used C6-glioma and B35-neuronal cells as model, investigated the interaction of MeHg and SeMet. Though efforts have been made on the study of effect of Se on MeHg cytotoxicity, all these works focus on the evaluation of biological parameters rather than the study of Hg-Se species and transformations which intuitively reveal the interaction between Se and MeHg at molecular level. Metallomics, proposed in 2004 by Prof. H. Haraguchi^23^, integrates the research fields related biometals, in which elemental speciation is the crucial strategy to elucidate the biological or physiological functions of biometals in the biological systems. It gives us a hint that the speciation study of Se against MeHg toxicity would provide new perspective in the research of Hg-Se interactions in physiological systems.

Hyphenated techniques such as liquid chromatography- inductively coupled plasma mass spectrometry (LC-ICP-MS) are the most effective methods for simultaneous speciation of Se and Hg in biological samples^24–26^. However, due to the limited amount of cell samples, low concentration of interested elemental species and complex cell matrix, miniaturized sample pretreatment is essential prior to elemental speciation^27–30^. Therefore, to study the interaction of MeHg-Se at cellular level, a systematic analytical platform is in great urgent.

In this work, to reveal the effect of selenocystine (SeCys_2_) against MeHg cytotoxicity in HepG2 cells, a comprehensive analytical platform for speciation study of mercury and selenium in MeHg incubated or MeHg + SeCys_2_ co-incubated HepG2 cells was developed by the integration of liquid chromatography (LC) - inductively coupled plasma mass spectrometry (ICP-MS) hyphenated techniques and chip-based pretreatment method. Results obtained by the developed comprehensive analytical platform provided a possible pathway for the incorporation and excretion of mercury species with the coexistence of SeCys_2_ at molecular level, and reveals the mechanism of SeCys_2_ against MeHg cytotoxicity in HepG2 cells.

## Results

**Figure 1:**
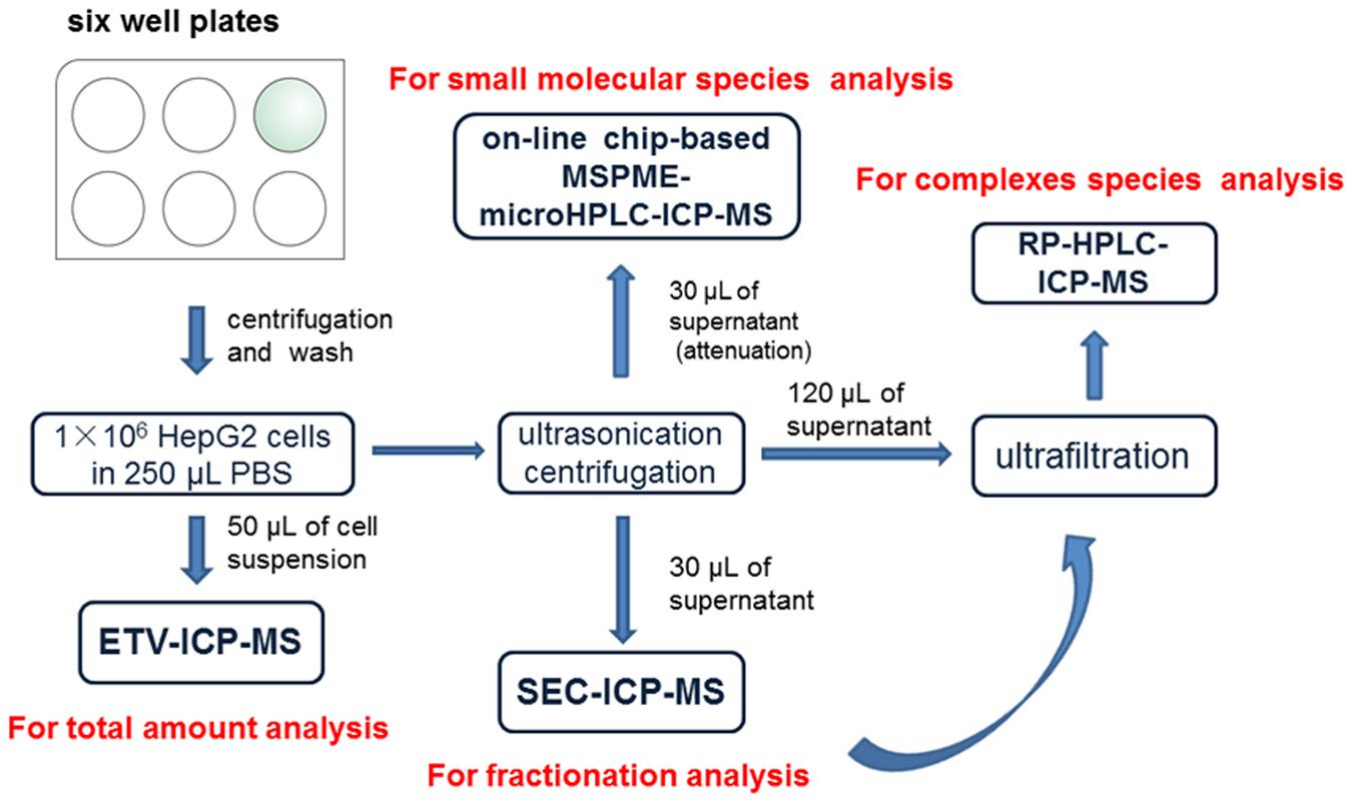
The diagram scheme for the comprehensive analytical platform by using ICP-MS based hyphenated techniques. ETV: electrothermal vaporization; SEC: size exclusion chromatography; RP-HPLC: reversed phase high performance liquid chromatography; MSPME: magnetic solid phase microextraction.

To investigate the species transformation of SeCys_2_ and MeHg in HepG2 cells, we constructed a comprehensive analytical strategy which is shown in Fig. 1. The total amounts of Hg and Se were measured by electrothermal vaporization (ETV)-ICP-MS, species of Hg and Se in cell cytosol was determined by size exclusion chromatography (SEC)-ICP-MS, reversed phase (RP) HPLC-ICP-MS and chip-based magnetic solid phase microextraction-microHPLC-ICP-MS. In addition, the possible species of Hg and Se in cell culture medium and cell elimination medium have also been studied to reveal the mechanism of SeCys_2_ against MeHg cytotoxicity.

### MTT assay

According to previous studies, a wider number of Se species, both inorganic and organic ones have been considered as the potential compounds to protect cells from MeHg cytotoxicity. In this work, inorganic Se species such as Se(IV) and organic Se species such as SeCys_2_, SeMet and MeSeCys were chosen as alternative species. Firstly, the cytotoxicity of these Se species and MeHg was investigated by MTT assay. The results are shown in Figure S1. According to the results, median lethal concentration (MLC) of MeHg is 13.0 μmol L^−1^, SeMet is nontoxic with the incubation concentration of 0–150 μmol L^−1^, SeCys_2_ and SeMeCys are mildly toxic when the incubation concentration is higher than 60 μmol L^−1^, and the MLC of Se(IV) is 97.6 μmol L^−1^.

In order to find out the most effective species to reduce the cytotoxicity of MeHg, four Se species were co-incubated with MeHg (15 μmol L^−1^) for 24 h at different concentration ratios, the cell viabilities are studied by MTT assay and the results are shown in Figure S2. As can be seen, the co-incubation of MeHg and SeCys_2_ significantly increases the cell viability which increases with the increase of the co-incubation concentration of SeCys_2_. The results demonstrate that SeCys_2_ is effective to against MeHg cytotoxicity. The following study will be focused on the interaction of SeCys_2_ and MeHg in HepG2 cells.

Since the low cell viabilities would make the cell collection from well plates difficult and introduce inaccuracy, in the following experiments, the co-incubation concentration of MeHg was chosen as 7.5 μmol L^−1^ to make sure that the cell viabilities for all the incubation conditions are higher than 85% (results shown in Figure S3).

### Total mercury and selenium in HepG2 cells

The effect of SeCys_2_ against MeHg cytotoxicity may root in the influence of MeHg uptake in HepG2 cells. Therefore, the total amount changes of Hg and Se in HepG2 cells should be investigated.

Traditional method for the determination of total Hg and Se by ICP-MS always involves acid digestion which consumes large number of cells (at last several million). In this work, an ETV-ICP-MS method was used for the determination of total Hg and Se in HepG2 cells. The analytical performance and the recoveries are shown in Table S1. The results of total Hg and Se in HepG2 cells incubated with MeHg or co-incubated with MeHg and SeCys_2_ are shown in Figure S4. The results demonstrated that the addition of SeCys_2_ increase rather than decrease the uptake amount of MeHg in cells. Therefore, the effect of SeCys_2_ against MeHg cytotoxicity should be further studied from the view of elemental species changes.

For further speciation analysis, the co-incubation concentration ratio of MeHg/SeCys_2_ was chosen to be 1:0, 3:1, 1:1 and 1:3 as representative. And the time course changes in total mercury and selenium are shown in Figure S5.

### Species of mercury and selenium in HepG2 cells

**Figure 2:**
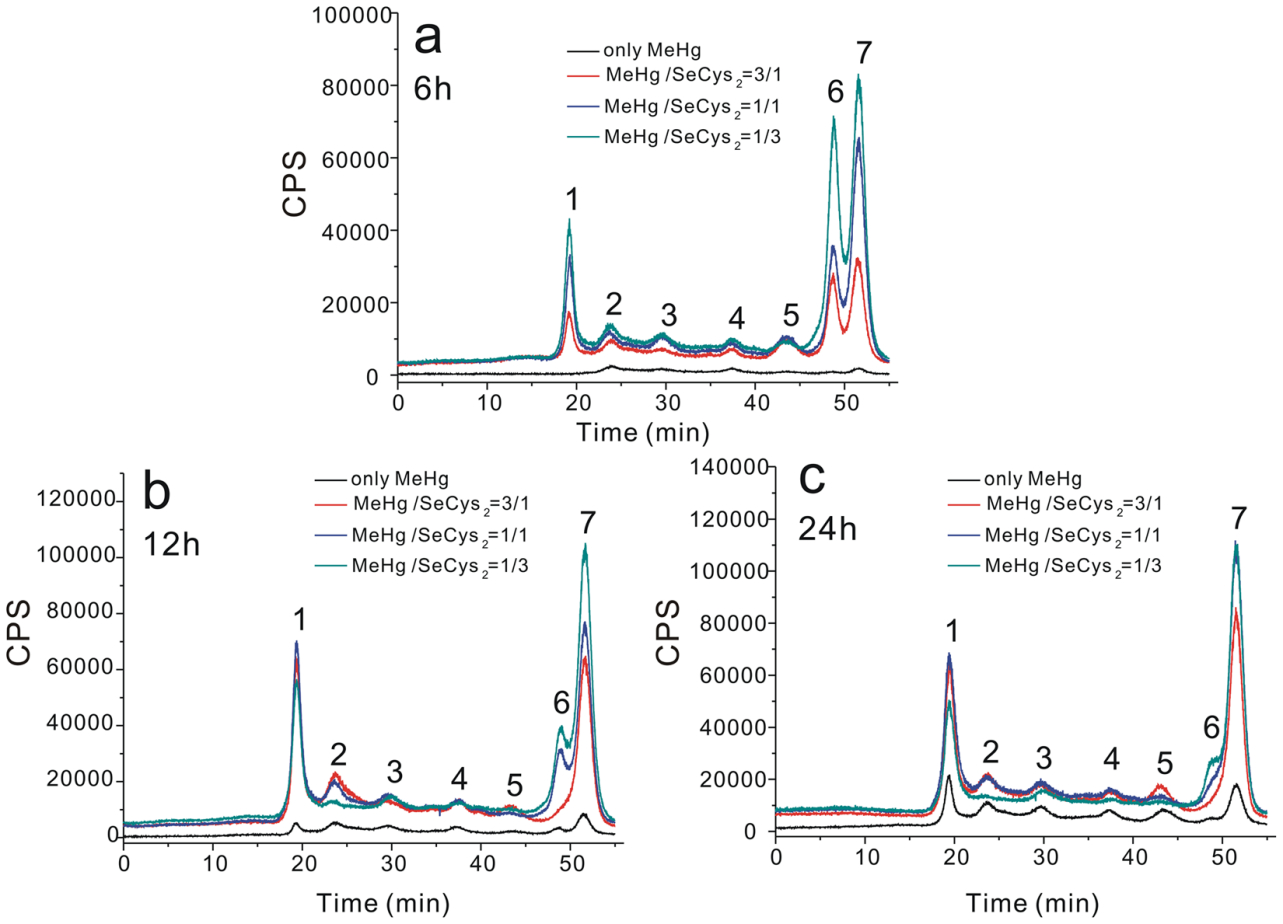
Chromatograms of mercury species obtained by SEC-ICP-MS for the cytosol of cells incubated with MeHg, and co-incubation with MeHg + SeCys_2_ at the ratios of 3:1, 1:1, 1:3 for 6 h (**a**), 12 h (**b**) and 24 h (**c**), respectively. (Information of molecular weight: fraction 1 > 669 kDa, 669 kDa > fraction 2 > 440 kDa, 440 kDa > fraction 3 > 67 kDa, 67 kDa > fraction 4 > 35 kDa, 35 kDa > fraction 5 > 6512 Da, fraction 6 and 7 < 1355 Da).

For the investigation of Hg and Se species in HepG2 cells, the cell cytosol was subjected to SEC-ICP-MS detection for initial fractionation based on molecular weights and the results are shown in Figs 2 and S6. The mercury species in cytosol could be classified into seven fractions, and fraction 1, 6 and 7 are the major species. The selenium species in cytosol could be classified into two major species (fraction 8 and 9). Overall, all the fractions increased with the increase of incubation ratio of the SeCys_2_ to MeHg, and all the fractions except fraction 6 increased when increasing incubation time. The changes of small molecules mercury species (fraction 6 and 7) in cell cytosol are the most dramatic, which might be the main reason leading to lesser cytotoxicity of MeHg. In the following study, we put our effort on these small molecules species.

**Figure 3:**
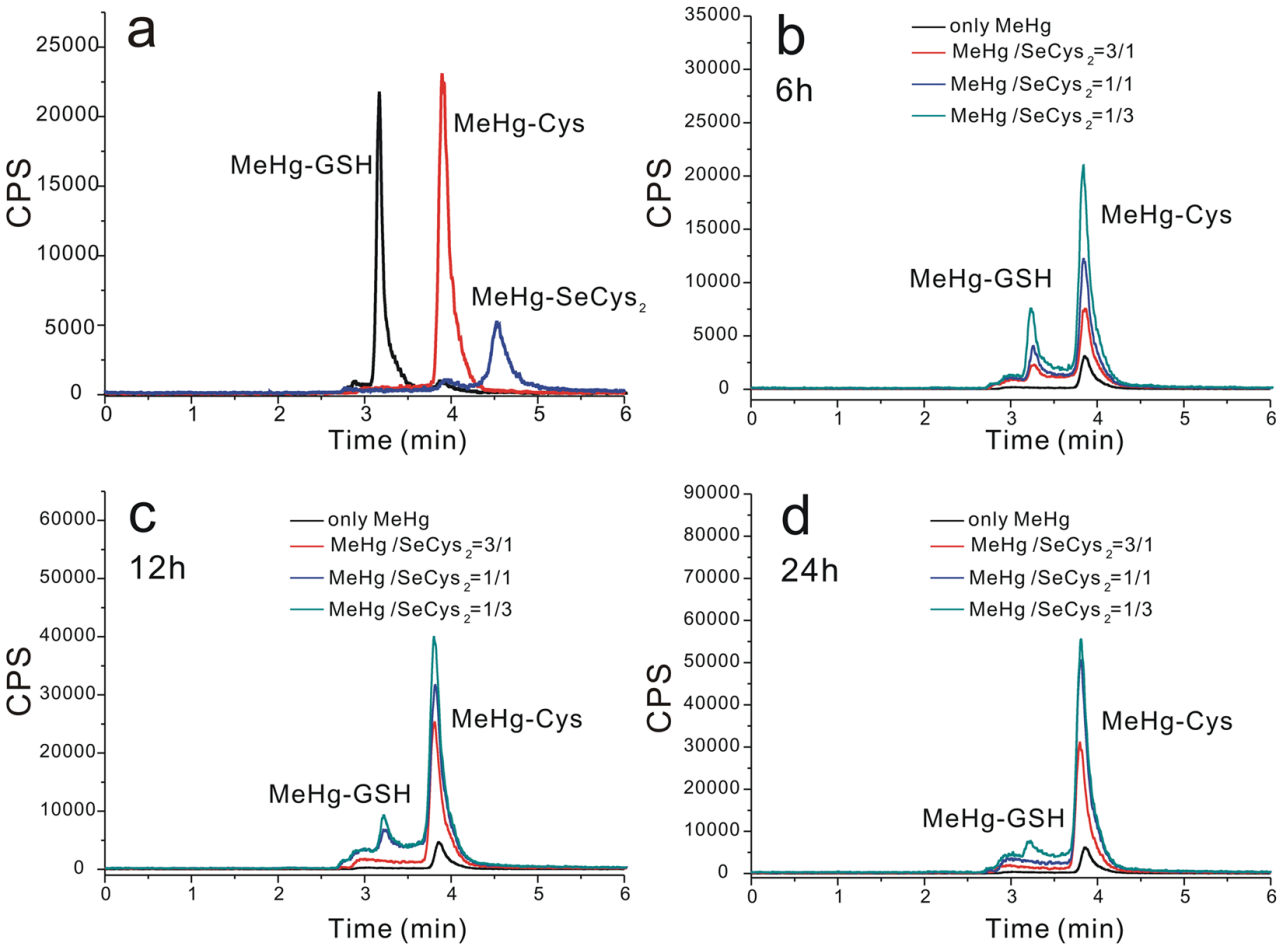
Chromatograms of mercury species obtained by RP-HPLC-ICP-MS in the standard solution of MeHg-GSH, MeHg-Cys and aggregation of MeHg and SeCys_2_, respectively (**a**) and in the cytosol of cells incubated with MeHg, and co-incubation with MeHg and SeCys_2_ at the ratios of 3:1, 1:1, 1:3 for 6 h (**b**), 12 h (**c**), 24 h (**d**), respectively.

Then the small molecule species of the cell cytosol were collected by ultrafiltration, and subjected to RP-HPLC-ICP-MS analysis. According to the retention time, the major mercury species were recognized as MeHg-GSH and MeHg-Cys as shown in Fig. 3 (their concentration are listed in Table S2), and the selenium species was recognized as SeCys_2_ as shown in Figure S7. As can be seen, the concentrations of MeHg-GSH and MeHg-Cys increase with the increase of incubation ratio of SeCys_2_ to MeHg, but the concentration of MeHg-GSH decreases with the increase of incubation time, which are in accordance with the variation of fraction 6 and 7 in SEC-ICP-MS analysis. Therefore, the standard solutions of MeHg-GSH, MeHg-Cys, and mixture of MeHg and SeCys_2_ (the aggregation of MeHg and SeCys_2_ has a mole ratio of Hg to Se as 4:1 and its structure was studied by electrospray ionization- quadrupole- time of flight- mass spectrometry (ESI-Q-TOF-MS) (Figure S8), matrix-assisted laser desorption ionization (MALDI)-TOF-MS, ^1^H NMR (Figure S9) and quantum mechanical calculation (Figure S10 and Table S3), seen in Supporting Information) were then subjected to SEC-ICP-MS analysis, and the retention times of MeHg-GSH and MeHg-Cys shown in Figure S6 are coincided with that of fraction 6 and 7, respectively, strongly demonstrating the MeHg-GSH and MeHg-Cys are the component of fraction 6 and 7, respectively.

Besides small molecules mercury complexes, the dissociative MeHg^+^ and the degradative Hg^2+^ were also studied by on-line chip-based MSPME-microHPLC-ICP-MS^31^. As shown in Table S4, the concentrations of MeHg^+^ and Hg^2+^ increase along with the increase of incubation time and incubation ratio of SeCys_2_ to MeHg, but the concentrations of them are very low.

### Species of mercury and selenium in culture medium

**Figure 4:**
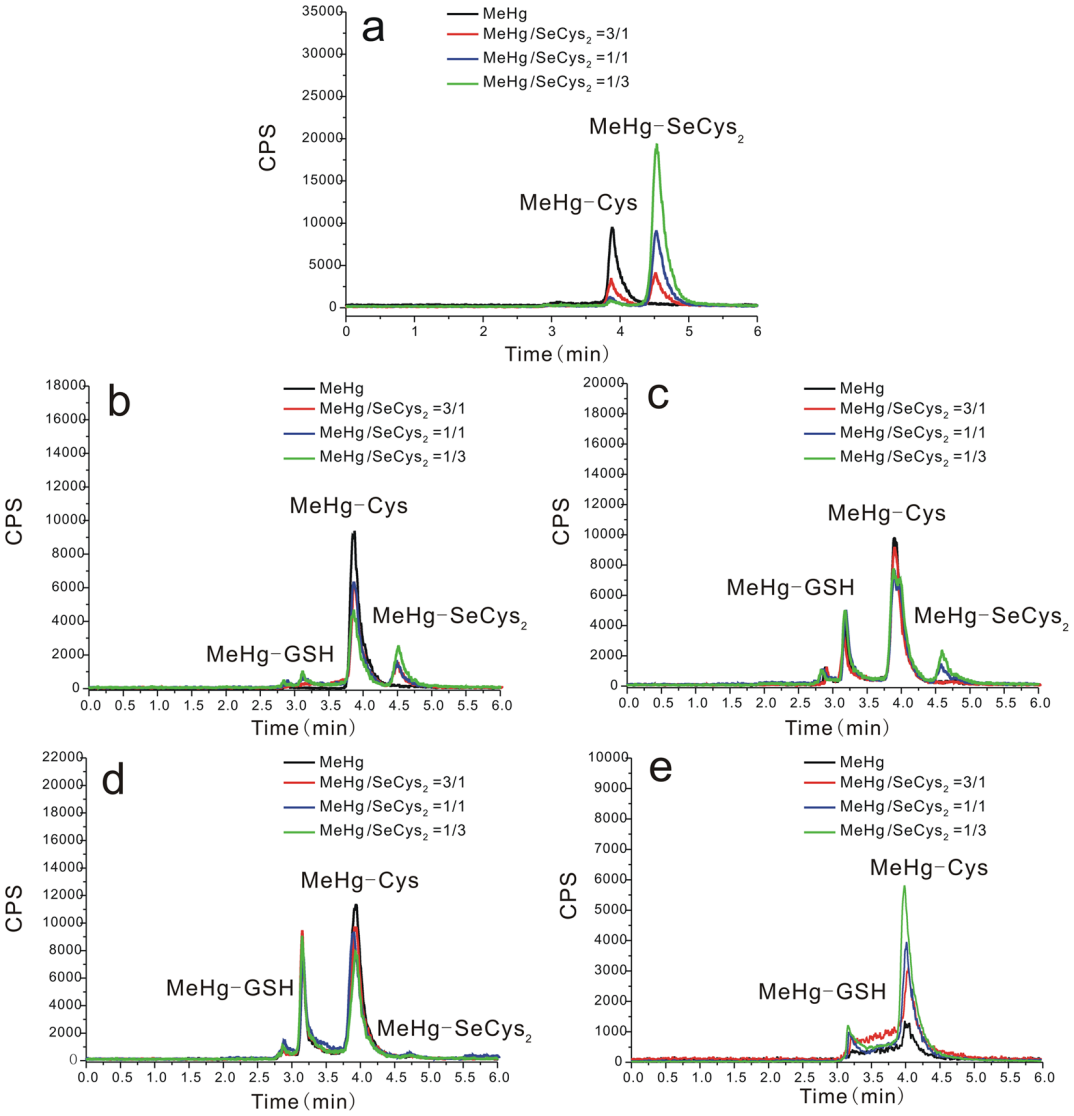
Chromatograms of mercury species obtained by RP-HPLC-ICP-MS. Mercury species in the culture medium without cells (**a**); mercury species in the culture medium with cells incubated with MeHg, and co-incubation with MeHg + SeCys_2_ at the ratios of 3:1, 1:1 and 1:3 for 6 h (**b**), 12 h (**c**) and 24 h (**d**), respectively; mercury species in the culture medium after elimination for 24 h (**e**).

To figure out whether these main species in cell cytosol are small molecules mercury complexes form in HepG2 cells or not, the analysis of Hg and Se species in cell culture medium was performed by RP-HPLC-ICP-MS. The results are shown in Fig. 4, Figure S11 and Table S5. As can be seen, there are three mercury species (aggregation of MeHg and SeCys_2_, MeHg-GSH and MeHg-Cys) in the cell culture medium. Aggregation of MeHg and SeCys_2_ could also be observed in culture medium without cells (Fig. 4a). Concentration of aggregation of MeHg and SeCys_2_ decreases along with the increase of the incubation time (Fig. 4b–d). The amount of MeHg-GSH (Fig. 4b–d) greatly increases with the increase of the incubation time. For MeHg-Cys, its amount (Fig. 4b–d) slightly decreases with the increase of the incubation ratio of SeCys_2_ to MeHg.

To investigate the elimination species of mercury from the HepG2 cells, the elimination medium was also studied. After changing the culture medium into a new one which did not contain MeHg and SeCys_2_, both MeHg-GSH and MeHg-Cys could still be found in the elimination medium (Fig. 4e), and their concentration increased with the increase of the incubation ratio of SeCys_2_ to MeHg.

## Discussion

The results of total mercury determination and toxicity test in HepG2 cells show an interesting phenomenon that the co-incubation of SeCys_2_ promoted the uptake of MeHg, but reduced the cytotoxicity of MeHg. It is reported that the co-administration of Cys with MeHg increased the uptake of MeHg^32^, and MeHg-Cys was one of the transportable substrates for cells^33^. And the generation of MeHg and SeCys_2_ aggregation may possess similar functionality. The decrease of aggregation of MeHg and SeCys_2_ in culture medium along with the incubation time demonstrated that aggregation of MeHg and SeCys_2_ is one of the transportable substrates of MeHg in cells and that is probably why the addition of SeCys_2_ increased the uptake of MeHg in HepG2 cells.

**Figure 5:**
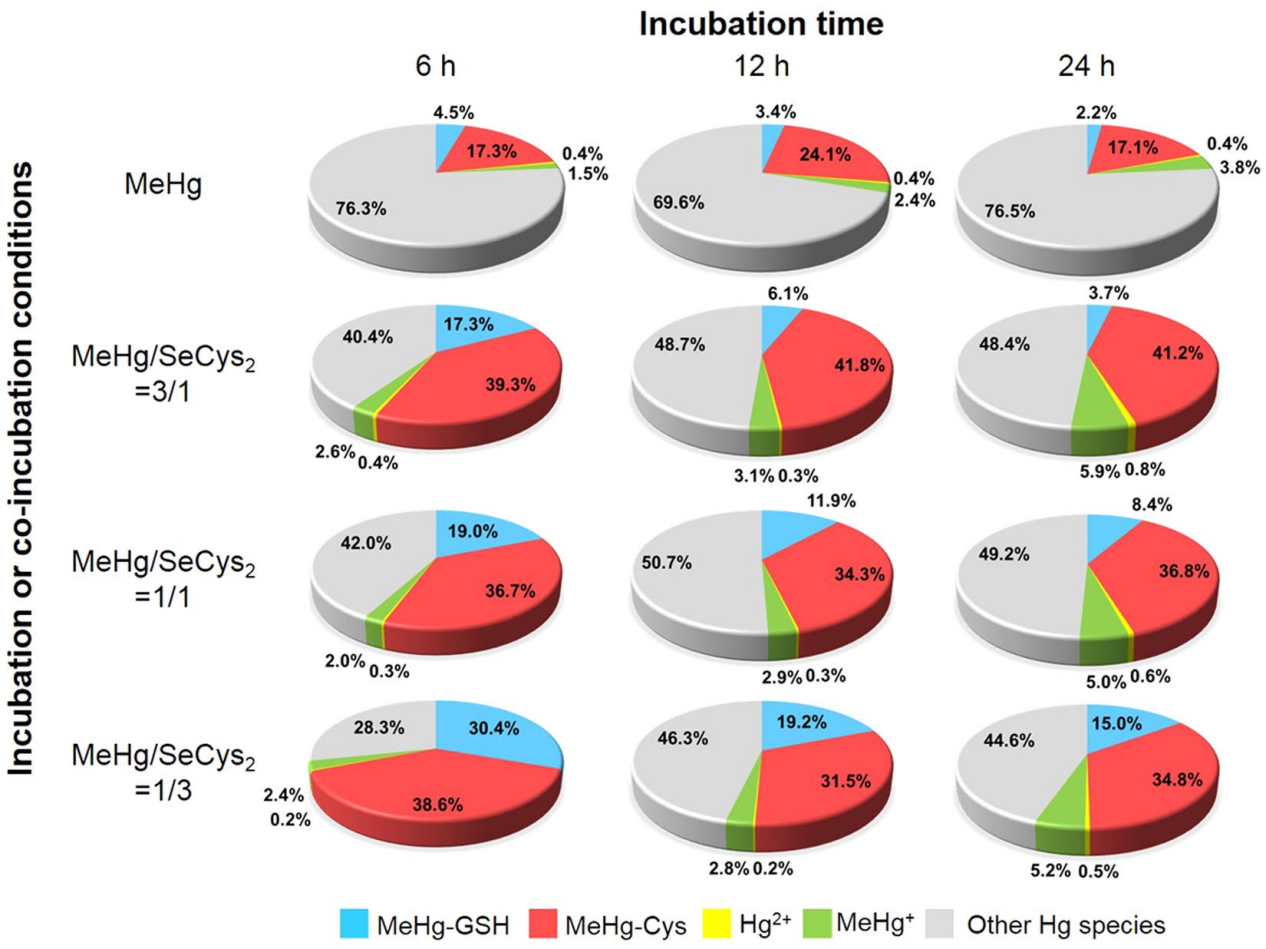
Proportions of MeHg-GSH, MeHg-Cys, Hg^2+^, MeHg^+^ and other species of mercury in a HepG2 cell. The total amounts of mercury were obtained by ETV-ICP-MS determination; the mount of Hg^2+^ and MeHg^+^ were obtained by chip-based on-line MSPME-microHPLC-ICP-MS analysis; the amount of MeHg-GSH and MeHg-Cys were obtained by C18 RP-HPLC-ICP-MS measurement; the normalization method was use for the calculation. (The values of 4.5%, 3.4%, 2.2%, 6.1%, 3.7% and 8.4% of MeHg-GSH were calculated based on the semi-quantified results because the concentrations of MeHg-GSH under these incubation conditions are lower than the LOQ but higher than the LOD by RP-HPLC-ICP-MS analysis).

The results of mercury species in cell cytosol obtained above are integrated and the proportions of different Hg species in one HepG2 cell were shown in Fig. 5. As can be seen, though the total Hg increased fiercely with the co-incubation of SeCys_2_, more proportion of them transfer into complexes species (MeHg-GSH and MeHg-Cys), this phenomenon may be the main reason to reduce the influence of MeHg on other physiological process. Besides, it can be confirmed that MeHg-GSH was formed in HepG2 cells because it was found in HepG2 cells, culture medium with HepG2 cells, but not in the culture medium without HepG2 cells.

The results for the speciation of mercury in elimination medium demonstrated that both MeHg-GSH and MeHg-Cys were the main mercury species. It was found in rat model experiment^34, 35^ that MeHg-GSH was the transformation species to transfer MeHg from liver to bile. Since MeHg-GSH was formed in HepG2 cells and was found in the elimination medium, MeHg-GSH is the species for cells elimination of MeHg. In addition, the concentration of MeHg-GSH dramatically increased along with the incubation time, which again implied that MeHg-GSH was the main elimination species to reduce the cytotoxicity of MeHg. MeHg-Cys was found in all culture medium including elimination medium with a high concentration. Since Cys is the component of culture medium with the concentration of 0.2 mM and the complex stability constants of MeHg-Cys (Logβ = 26.05) is relatively high, a complex reaction between MeHg and Cys or a competition reaction between MeHg complexes with Cys would occur in the culture medium.

In summary, this study on SeCys_2_ against MeHg cytotoxicity demonstrates a possible pathway for the incorporation and excretion of mercury species at molecular level, and reveals the mechanism of SeCys_2_ against MeHg cytotoxicity in HepG2 cells: aggregation of MeHg and SeCys_2_ is the main species to promote the uptake of MeHg, MeHg-GSH is the species for the elimination of MeHg from HepG2 cells, the transformation of MeHg into complexes species (MeHg-GSH and MeHg-Cys) in cytosol is the key point for the detoxication of SeCys_2_ to MeHg cytotoxicity in HepG2 cells.

## Methods

The details of apparatus, reagents, standard solutions, cell culture and MeHg treatment could be found in Supporting Information. The Optimized operating conditions for online HPLC-ICP-MS and ETV-ICP-MS are listed in Tables S6 and S7, respectively.

### Cell viability assay

In cell viability assay, the cells were seeded on 96 well plates with a number of 10,000 per well, after grown for 24 h the cells were incubated with MeHg, Se(IV), Se(VI), SeCys_2_, SeMet and MeSeCys, respectively, or co-incubation with MeHg and different Se species (including Se(IV), SeCys_2_, SeMet and MeSeCys) for 24 h. Then the solution was removed, the cells were incubated with MTT solution (0.5% (m/v)) for 4 h. After that, the MTT solution was removed and DMSO was added allowing for vibration for 10 min, and the absorbance was measured using a microplate reader at 570 nm.

### Cell samples preparation

For the study of SeCys_2_ against MeHg cytotoxicity in HepG2 cells, the HepG2 cells cultured with 10% fetal bovine serum in DMEM medium are used as blank samples (termed as blank groups), while the HepG2 cells incubated with MeHg are used for comparison (termed as control groups, cell viability 86.7% shown in Figure S3).

The detail of cell sample preparation was shown in Fig. 1. The cells in the wells were treated with centrifugation (1200 rpm, 3 min) after cell dissociation, the supernatant was removed and the cells were washed with PBS twice. Then the amount of cells was counted, and the density of cell suspension solution was diluted to 1 × 10^6^ HepG2 cells in 250 μL PBS.

50 μL of this suspension was attenuated to 1,000 cells per 7 μL for the cells co-incubated with MeHg and SeCys_2_, while the density of cells incubated with MeHg was fixed to 5,000 cells per 7 μL. And then the attenuated cell suspension was subjected to ETV-ICP-MS for the detection of total Hg and Se.

The rest cell suspension solution (200 μL) was subjected to ultrasonication, 40 times a circle, 30 s per time, 30 s intervals for each time. Then the cell suspension solution was treated with centrifugation at 12,000 rpm for 10 min. After that, the supernatant was separated into three portions: portion 1 (30 μL), portion 2 (120 μL) and portion 3 (30 μL). Portion 1 (120,000 cells per sample) was introduced into SEC-ICP-MS for the speciation of Hg and Se in cell cytosol. Portion 2 was treated with ultrafiltration (3 KDa) and introduced into C18 RP-HPLC-ICP-MS directly (injection volume 30 μL, 120,000 cells per samples). For portion 3, it was diluted by 40 times and then subjected to the chip-based on-line MSPME-microHPLC-ICP-MS analysis for the determination of dissociative Hg species (10,000 cells per sample). The operation of chip-based on-line MSPME-microHPLC-ICP-MS was described in our previous work^31^.

The possible Hg and Se species in the culture medium: the culture medium with the cells incubated with MeHg and co-incubated with MeHg + SeCys_2_, at 6, 12 and 24 h, respectively, and the elimination culture medium were analysed by C18 RP- HPLC-ICP-MS, as well.

### Statistical analysis

Data of MTT assay are presented as means ± standard deviation for six replicate measurements. Data of ETV-ICP-MS determination are presented as means ± standard deviation for five replicate determinations. Data of C18 RP-HPLC-ICP-MS determination are presented as means ± standard deviation for triplicate analysis. Data of chip-based on-line MSPME-microHPLC-ICP-MS analysis are presented as means ± standard deviation for triplicate analysis.

All results were analyzed by SPSS Statistics as follows: The data of samples were tested by non-parameter method, and Friedman test was used for the data analysis with *p* ≤ 0.05. The data of control and experiment groups were tested by paired sample T-test, and results showed that there are significant differences between control group and experiment groups with *p* ≤ 0.05.

## Electronic supplementary material

Supplementary Info File #1
