# Supplementary material for: Selenocystine against methyl mercury cytotoxicity in HepG2 cells
Source: Sci Rep. 2017 Mar 10;7:147. doi: 10.1038/s41598-017-00231-7 (PMC5428050; doi:10.1038/s41598-017-00231-7)
Supplement: Supplementary file 1 — Supplementary Info File #1 [file 41598_2017_231_MOESM1_ESM.doc]

**Supporting Information**

Selenocystine against methyl mercury cytotoxicity in HepG2 cells

Han Wang, Beibei Chen*, Man He, Xiaoxiao Yu, and Bin Hu*

Key Laboratory of Analytical Chemistry for Biology and Medicine (Ministry of Education), Department of Chemistry, Wuhan University, Wuhan, Hubei, 430072 (P.R.China)

Contact information: binhu@whu.edu.cn; bbchen@whu.edu.cn

**Apparatus**, **reagents and standard solutions.**

An X Series II ICP-MS (Thermo Fisher Scientific, USA) and a Dionex ultimate 3000 HPLC (Dionex, Germering, Germany) were used for speciation analysis in this work. SEC column (GE Healthcare Life Sciences, Superdex 200 10/300 GL, USA), microRP-C18 column (Dionex, C18 Acclaim, 3 µm, 300Å, 1.0 mm i.d.) and RP-C18 column (Elitehplc, Hypersil ODS2, 5 µm, 4.6×250 mm) were used for speciation analysis. The operation conditions for SEC-ICP-MS, on-line microHPLC-ICP-MS and RP-HPLC-ICP-MS are summarized in Table S5. An Agilent 7500a ICP-MS (Agilent, Tokyo, Japan) was equipped with a modified commercial WF-4C graphite furnace (Beijing Second Optics, Beijing, China) as an electrothermal vaporizer with a laboratory-built connection interface. And the operation conditions for ETV-ICP-MS are summarized in Table S6. A microTOF-Q III MS with an ESI ion source (Bruker, Germany), 5800 MALDI-TOF/TOF MS (AB SCIEX, USA), and ^1^H NMR (WNMR-I-400MHz, Varian, USA) were used for the structure study of the aggregation of MeHg and SeCys_2_. TS2-60 syringe pumps (Baoding Longer Precision Pump Co., Ltd., Baoding, China) and sterile syringes were used in chip-based magnetic solid phase microextraction system. An XS105 balance (Mettler Toledo Instruments Co., Ltd., Shanghai, China) was used for reagents weighing. A Legend 21R centrifuge (Thermo, USA) and ultrafiltration device (Millipore, Amicon Ultra, 3 KDa) were used for small molecules species collection in cell cytosol.

Stock solution (1 mg mL^-1^) of MeHg was prepared from analytical reagent grade of MeHgCl (Acros, Geel, Belgium) in methanol, stock solution (0.1 mg mL^-1^) of MeHg for cell incubation was prepared in deionized water. A stock solution (1 mg mL^-1^ as Se) of SeCys_2_ was prepared by dissolving L-selenocystine (98%, Acros Organics, USA) in 0.01 mol mL^-1^ of HNO_3_. Stock solutions (1 mg mL^-1^ as Se) of SeMet, MeSeCys and Se (IV) were prepared by dissolving DL-selenomethionine (99+%, Acros Organics, USA), Se-methylseleno-L-cysteine (98%, Acros Organics, USA) and Na_2_SeO_3_ (97%, Wako, Japan) in deionized water, respectively. All stock solutions were stored in the dark and refrigerator at 4 ºC. Working solutions were prepared daily using appropriate dilutions of the stock solutions. The phosphate buffer solution (PBS, pH 7.4) consists of 0.01 mol L^-1^ phosphate-buffered saline, and the reagents used were at least analytical reagent grade. Highly pure deionized water obtained from a Milli-Q system (18.2 MΩ·cm, Millipore, Molsheim, France) was used throughout the work. Dulbecco’s Modified Eagle Medium (DMEM) and fetal bovine serum (FBS) was obtained from Gibco Invitrogen Corporation (Thermo Fisher Scientific Inc., Waltham, MA, USA) and used for cell culture.

Methyl mercury complexes of MeHg-Cys and MeHg-GSH was obtained as follows: MeHgCl (5 µmol L^-1^) was mixed with Cys and GSH, respectively, at the mole ratio of 1/5 in PBS and then stirred for 3.5 h^1^. According to the complex stability constants of MeHg-Cys (Logβ=26.05) and MeHg-GSH (Logβ=21.77)^2^, the concentration of dissociative MeHg in PBS could be ignored, so the concentration of MeHg-Cys and MeHg-GSH are 5 µmol L^-1^ in the solutions. For the aggregation of MeHg and SeCys_2_, the complex at the mole ratio of 1/5, 1/20 and 1/100 were obtained and introduced into HPLC-ICP-MS to investigate the coordination equilibrium of MeHg and SeCys_2_. The results showed that at the mole ratio of 1/5, the concentration of dissociative MeHg in PBS could be ignored, so the concentration of MeHg and SeCys_2_ aggregation is 5 µmol L^-1^ in the solutions.

**Cell culture and methyl mercury treatment**

# Human hepatoma HepG2 cells (purchased from Stem Cell Bank, Chinese Academy of Sciences, Shanghai, China) were cultured in a humidified incubator containing 5% CO2 and 95% air at 37 ºC by DMEM medium, supplemented with 10% fetal bovine serum (FBS).

HepG2 cells were then seeded on six well plates with a number of 1×10^6^ per well, and incubated with MeHg (7.5 µmol L^-1^) or co-incubated with MeHg and SeCys_2_ at different concentrations (7.5 µmol L^-1^ MeHg+2.5 µmol L^-1^ SeCys_2_, 7.5 µmol L^-1^ MeHg+7.5 µmol L^-1^ SeCys_2_, 7.5 µmol L^-1^ MeHg+22.5 µmol L^-1^ SeCys_2_) for 6, 12 and 24 h, respectively.

The elimination experiment was also conducted. After the cells were incubated with MeHg or co-incubated with MeHg and SeCys_2_ for 24 h, the culture medium was changed with a new one which did not contain MeHg or SeCys_2_ for further incubation of cells for another 24 h.

**Blank control**

All the experiments were performed in a super-clean laboratory (ten-thousand class), and all the laboratory ware was made of polyethylene or Teflon material and thoroughly cleaned by soaking in 10% (v/v) nitric acid for at least 24 h or disposable.

The blank experiments were carried out as the method described above by using PBS buffer solution instead of the cell suspensions or the standard solutions. And all the analytical results were obtained by subtracting the blank values from the determined values.

**Structural study of MeHg and SeCys_2_ aggregation**

For the RP-HPLC-ICP-MS analysis, a new peak containing both Hg and Se with the retention time at 4.6 min was observed in the mixture of MeHg and SeCys_2_ when compared with the standard solution of MeHg and SeCys_2_. The concentration of Hg and Se for this peak were calculated by external calibrations, and it was found that the mole ratio of Hg to Se is 4:1. What is more, this mole ratio stayed the same in all conditions, indicating that it might a specific species. To identify this species, ESI-QTOF-MS (in both positive and negative mode), MALDI-TOF-MS, ^1^H NMR and quantum mechanical calculation were applied.

The standard solution of SeCys_2_ and the mixture of MeHg and SeCys_2_ at a ratio of 1:5 (10 mg L^-1^ as Hg) were analyzed by ESI-QTOF-MS in both positive and negative mode. As can be seen in Figure S8, the mass isotope pattern ascribed to SeCys_2_ was observed for the standard solution of SeCys_2_ and the mixture of MeHg and SeCys_2_, while no other species including MeHg were observed in the mixture solution of MeHg and SeCys_2_ (due to the poor sensitivity of ESI-QTOF-MS for mercury species). The results implied that no chemical bond was formed between MeHg and SeCys_2_.

For MALDI-TOF-MS analysis, no peak was found referred to the conjugation of MeHg and SeCys_2_. This result also indicated that there is no chemical bond between MeHg and SeCys_2_.

The ^1^H NMR analysis results for MeHg, SeCys_2_, the mixture of MeHg and SeCys_2_ were shown in Figure S9. Compared with the chemical shifts of MeHg and SeCys_2_, there is no change after MeHg was mixed with SeCys_2_, which also indicated that no chemical bond was formed between MeHg and SeCys_2_.

However, we did find an unknown species containing both Hg and Se by RP-HPLC-ICP-MS (retention time at 4.6 min) in the mixture of MeHg and SeCys_2_ (Figure 3a), in the culture medium without cells and the culture medium with cells incubated with MeHg, and co-incubation with MeHg and SeCys_2_ (Figure 4a-d and Figure S11a-d). So we hypothesized that there is a weak interaction between MeHg and SeCys_2_. To prove the hypothesis, quantum mechanical calculation was applied for the structural study and the results were shown in Figure S10 and Table S3. Geometrical optimizations were carried out using the density functional theory with the gradient-corrected functionals BLYP^3,4^ and the double numerical basis sets plus polarization^5^. Effective Core Potentials have been employed for both Se and Hg for relativistic corrections. The conductor-like screening model^6^ was used to simulate the aqueous solvent environment. Vibrational frequencies were calculated at the same level of theory to validate the nature of the optimized minima and to obtain the zero-point corrections and free energies. All the calculations were performed using the Dmol^3^ programs. As can be seen from Figure S11, MeHg^+^ could interact with SeCys_2_ via O in COO, N in NH_2_, and Se in SeCys_2_. The calculated bonding distances for COO...Hg, N...Hg, and Se...Hg are 2.2, 2.3, and 2.7 Å, respectively. And the calculated bond energy summarized in Table S7 indicated COO...Hg and N...Hg are the stable state for aggregation of MeHg and SeCys_2_, while Se...Hg is about 2/3 that of COO...Hg. Since SeCys_2_ has two carboxyl groups and amino groups, it can well explain that MeHg could aggregated with SeCys_2_ with the mole ratio of 4:1.

Taking into consideration of the results obtained by ESI-Q-TOF-MS/MS, MALDI-TOF-MS, ^1^H NMR along with quantum mechanical calculation, we hypothesized that MeHg interacted with SeCys_2_ to form MeHg and SeCys_2_ aggregation due to a weak interaction between MeHg and SeCys_2_. Some further works should be done to reveal the interaction mechanism between MeHg and SeCys_2_ and the structure of the MeHg and SeCys_2_ aggregation.

**Figure S1** Effect of MeHg, SeCys_2_, SeMet, MeSeCys and Se(IV) on cell viability. HepG2 cells were incubated with different concentrations of MeHg, SeCys_2_, SeMet, MeSeCys and Se(IV), respectively, for 24 h (means±s.d., n=6, error bars present the standard deviation of six parallel analysis).

**Figure S2** Effect of SeCys_2_, SeMet, MeSeCys and Se(IV) on the cytotoxicity of MeHg. HepG2 cells were co-incubated with MeHg and different concentrations of SeCys_2_, SeMet, MeSeCys and Se(IV), respectively, for 24 h (means±s.d., n=6, error bars present the standard deviation of six parallel analysis).

**Figure S3** Viability of cells incubated with 7.5 µmol L^-1^ MeHg and different concentrations of SeCys_2_ for 24 h (means±s.d., n=6, error bars present the standard deviation of six parallel analysis).

**Figure S4** The total Hg and Se in cells incubated with MeHg and SeCys_2_ at different ratios obtained by ETV-ICP-MS analysis (means±s.d., n=5, error bars present the standard deviation of five parallel analysis).

**Figure S5** Time course changes in total mercury and selenium in cells incubated with MeHg and SeCys_2_ at different ratios obtained by ETV-ICP-MS analysis (means±s.d., n=5, error bars present the standard deviation of five parallel analysis).


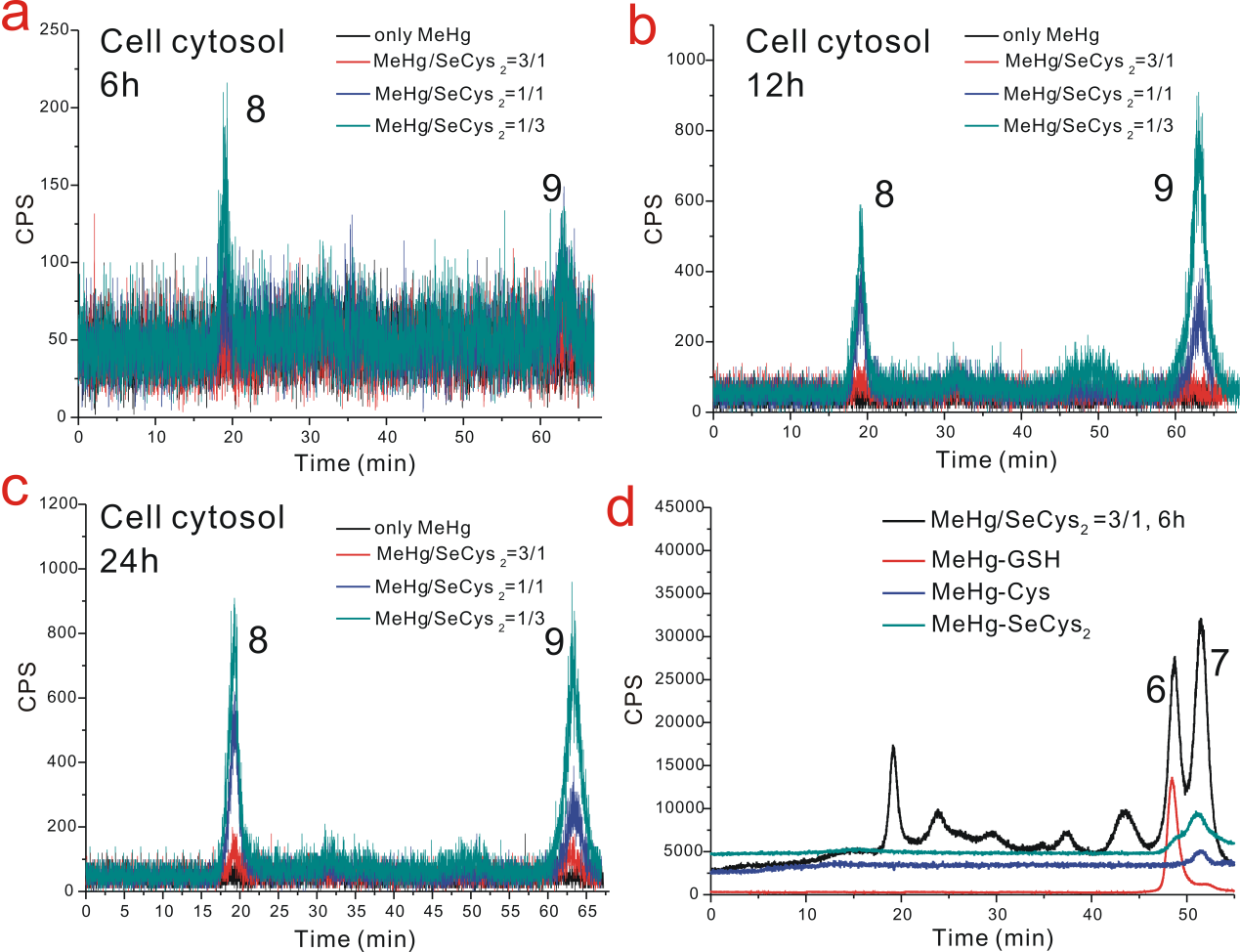


**Figure S6** Chromatograms of selenium species obtained by SEC-ICP-MS for the cytosol of cells incubated with MeHg, and co-incubation with MeHg and SeCys_2_ at the ratios of 3:1, 1:1, 1:3 for 6 h (a), 12 h (b), and 24 h (c), respectively, (information of molecular weight: fraction 8 > 669 kDa, fraction 9 < 1355 Da). Chromatograms of mercury species obtained by SEC-ICP-MS for three kinds of MeHg complexes (MeHg-GSH, MeHg-Cys, MeHg and SeCyes_2_ aggregation), and the chromatogram of mercury species obtained by SEC-ICP-MS for cytosol of cells co-incubated with MeHg and SeCys_2_ at the ratios of 3:1 for 6 h (d).


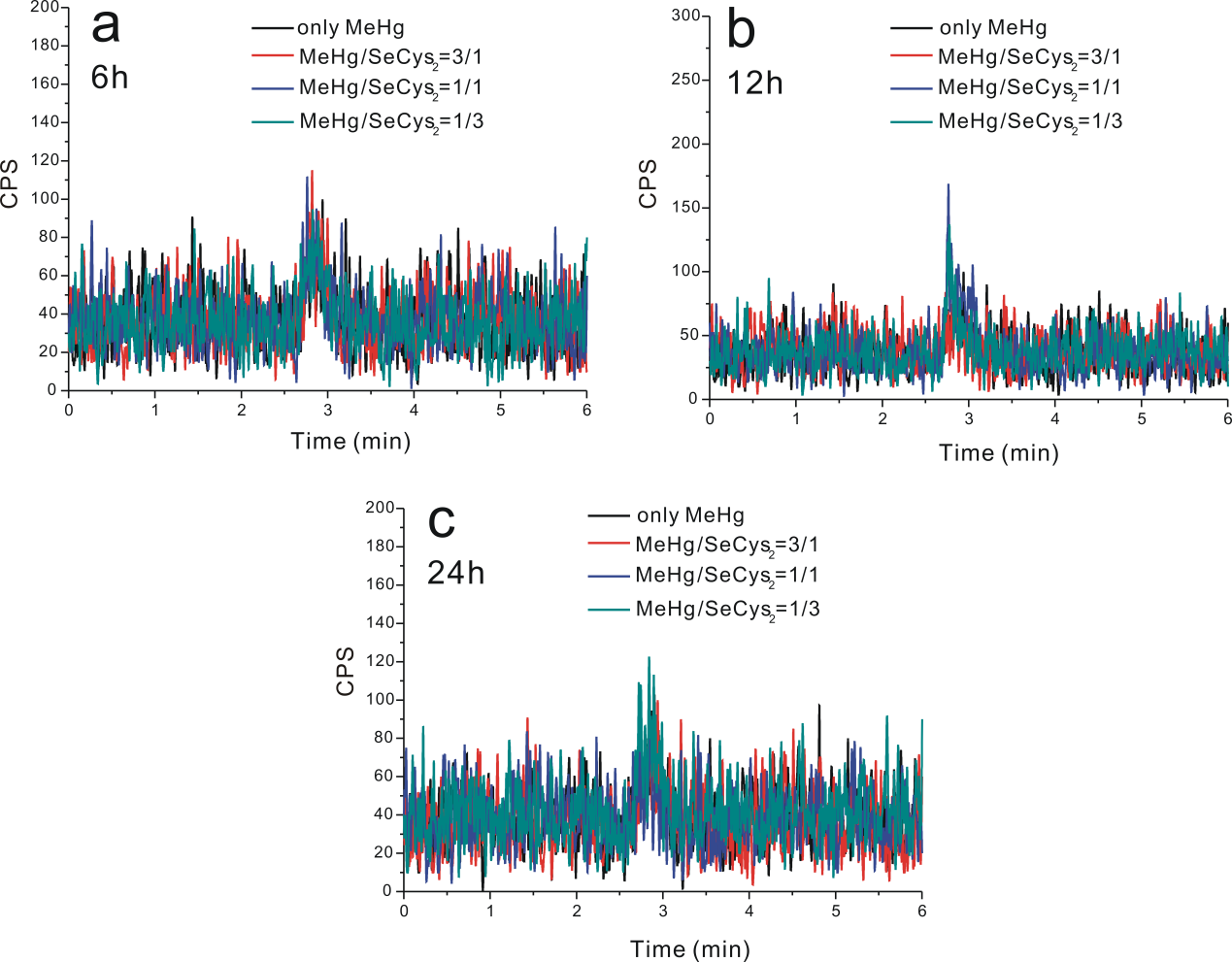

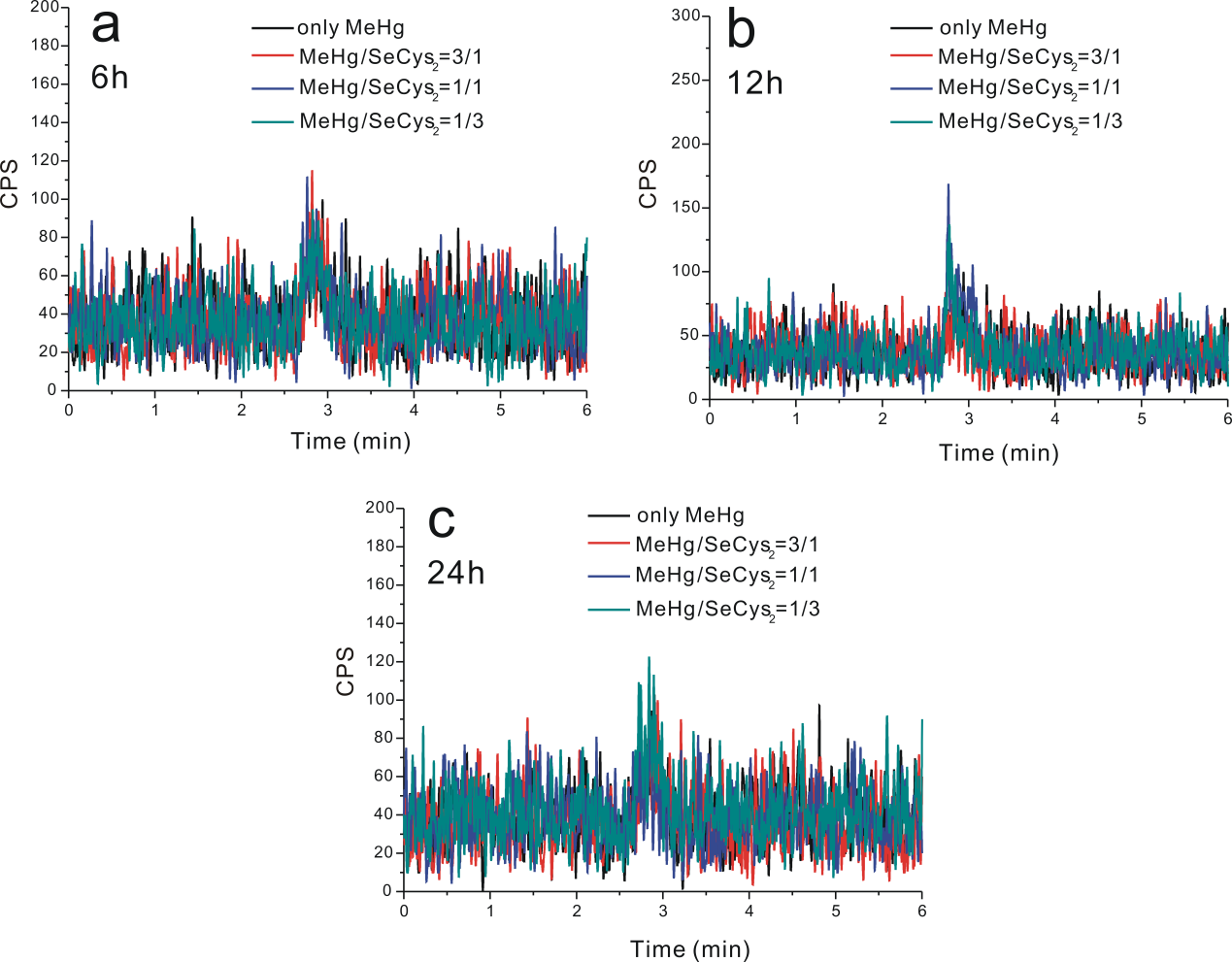

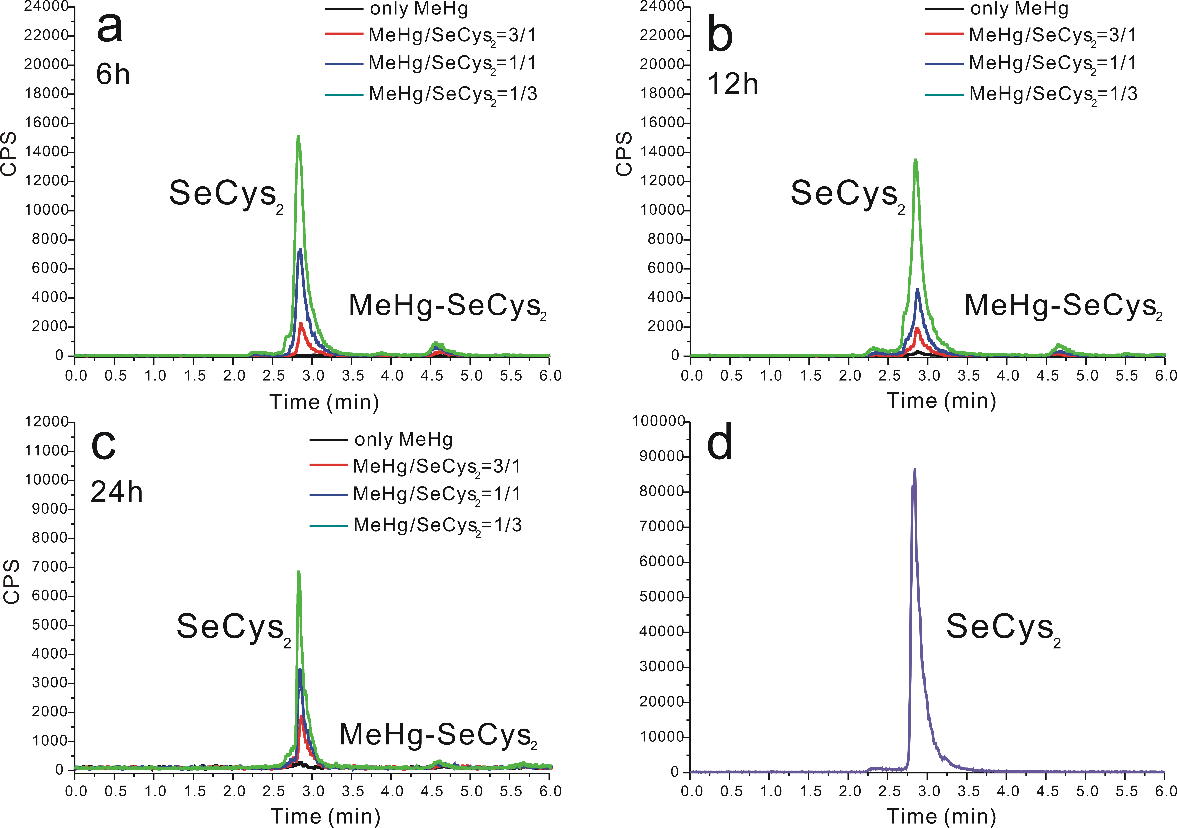


**Figure S7** Chromatograms of selenium species obtained by RP-HPLC-ICP-MS for the cytosol of cells incubated with MeHg, and co-incubation with MeHg and SeCys2 at the ratios of 3:1, 1:1, 1:3 for 6 h (a), 12 h (b), 24 h (c), respectively. And Chromatogram of SeCys_2_ obtained by RP-HPLC-ICP-MS (d).


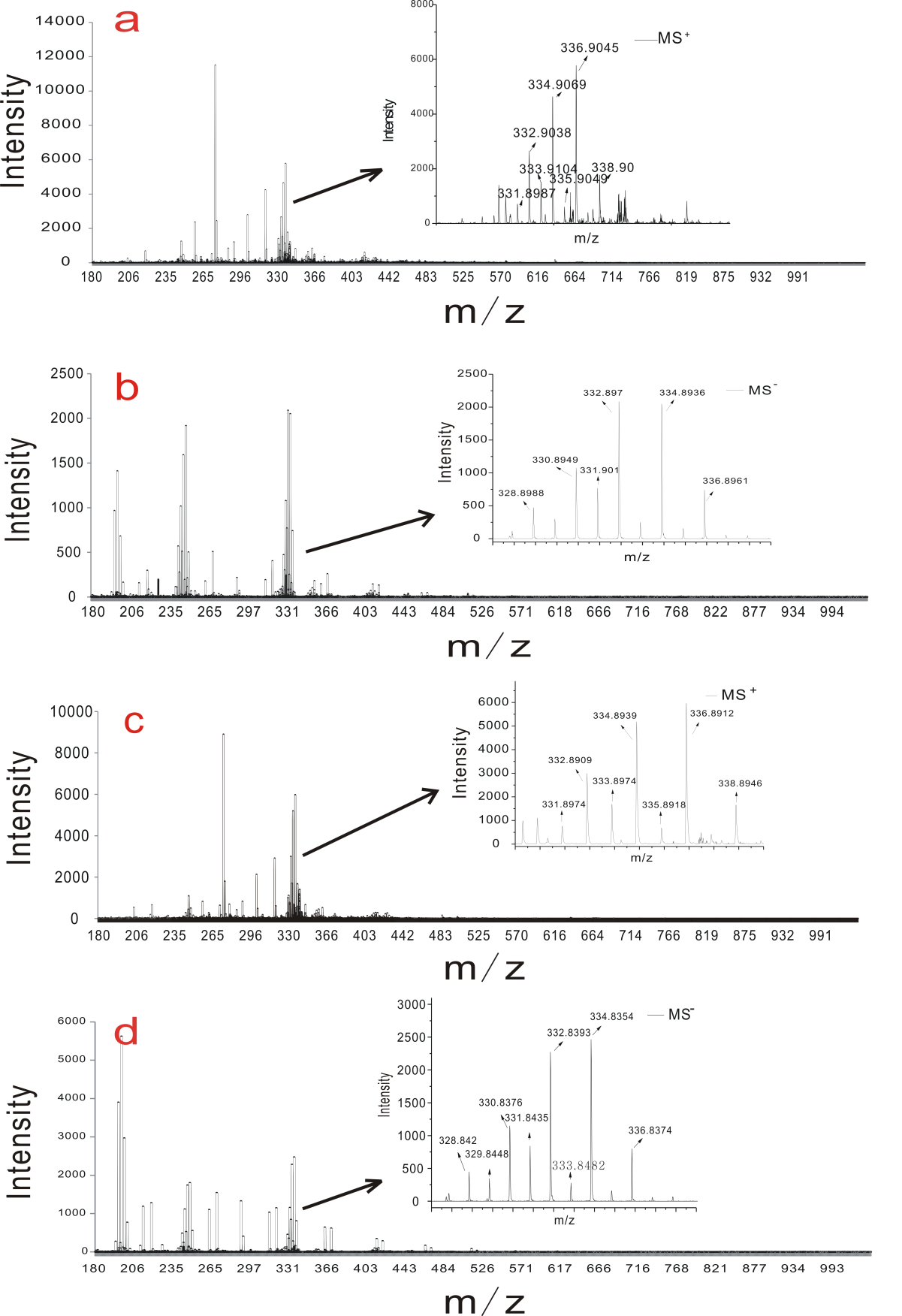


**Figure S8** ESI-QTOF-MS spectra of MeHg and SeCys_2_ in the mixture solution with positive mode (a), negative mode (b) and SeCys_2_ standard solution with positive mode (c), negative mode (d). SeCys_2_ (m/z=336.9, 334.9) was found in both the mixture solution of MeHg and SeCys_2_ and SeCys_2_ standard solution.


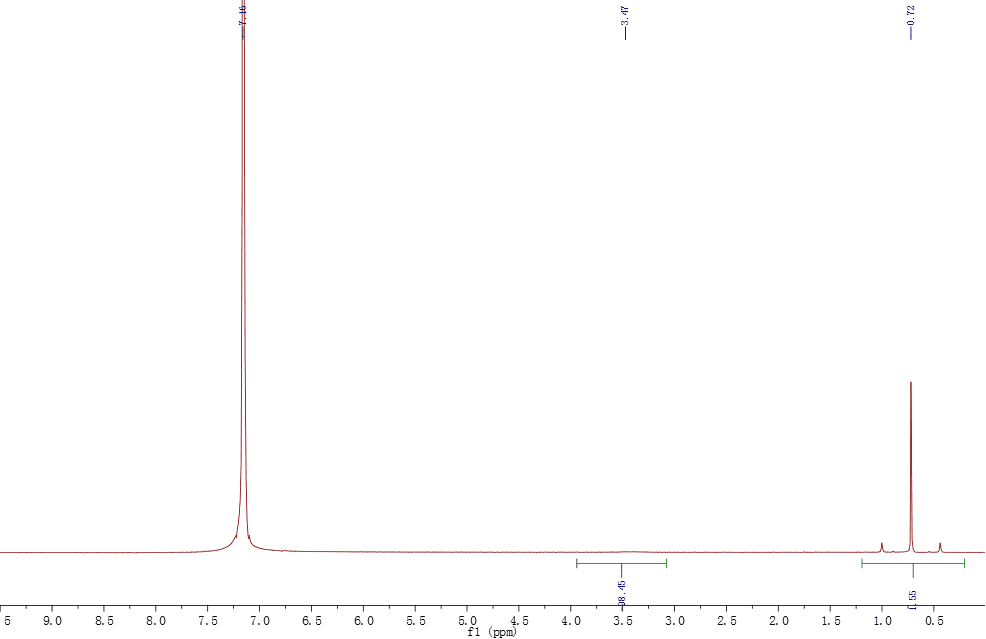

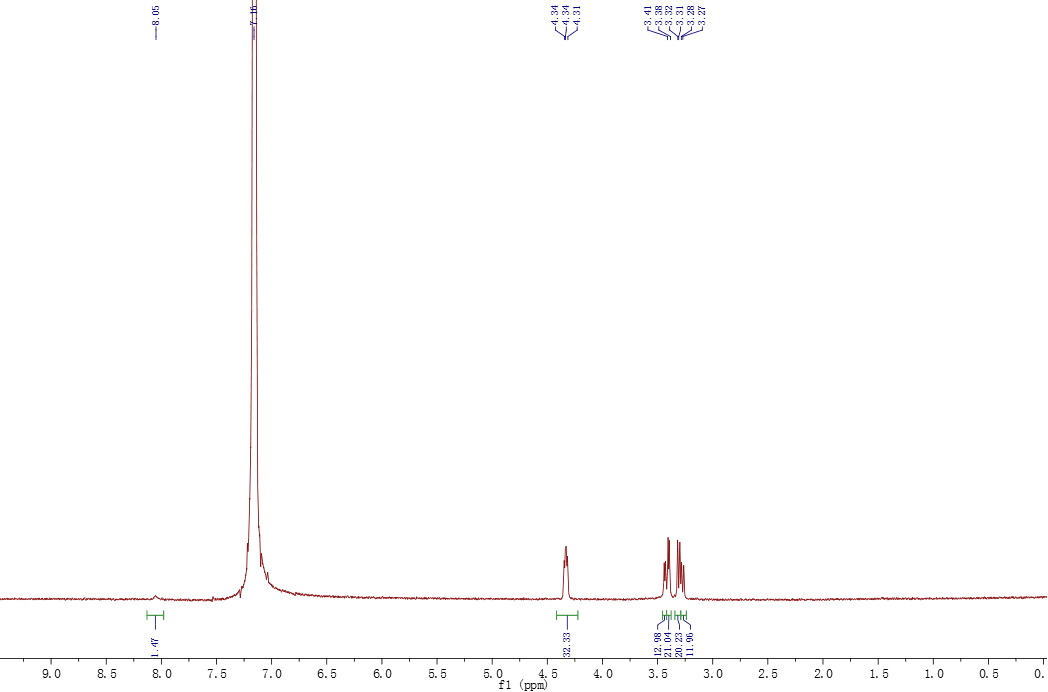

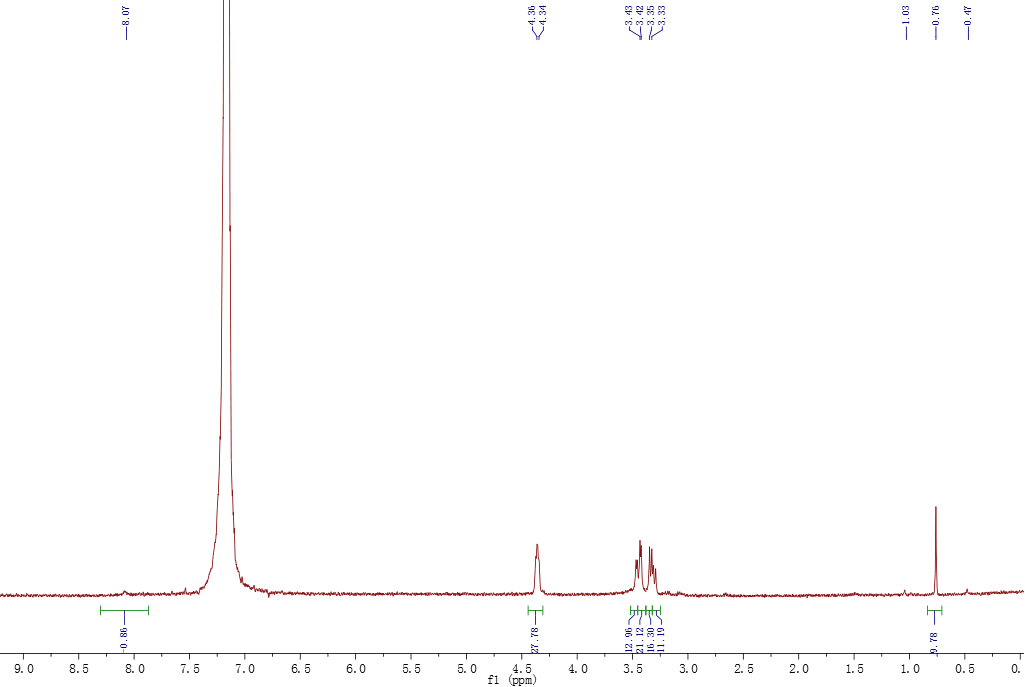


a

b

c

**Figure S9** ^1^H NMR spectra of MeHg (a), SeCys_2_ (b), and the mixture of MeHg and SeCys_2_ (c)


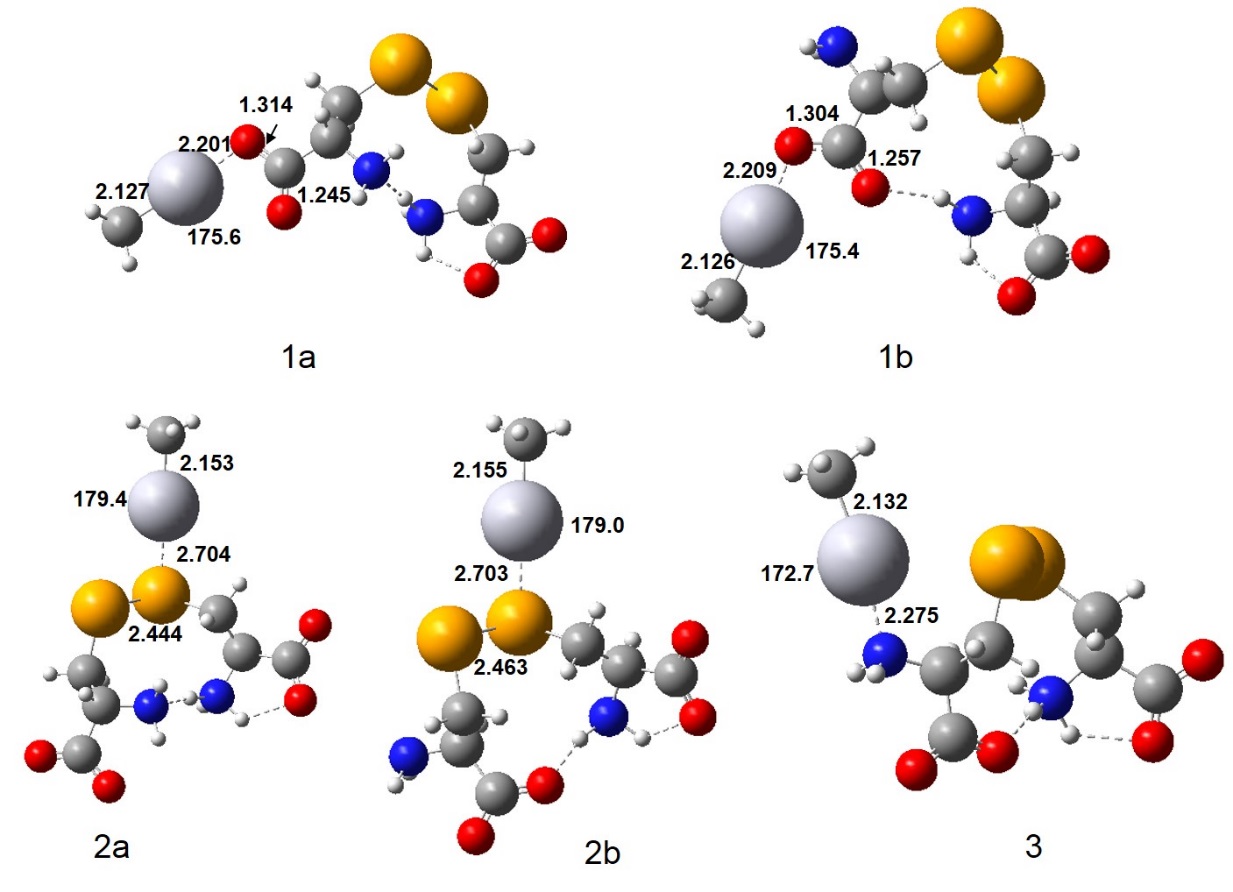


**Figure S10** Structures of five possible structures of MeHg and SeCys_2_ coordination. The structures were shown as ball-and-stick model, yellow balls represent Se, blue balls represent N, red balls represent O, grey balls represent C, big light gray balls represent Hg and small white balls represent H.


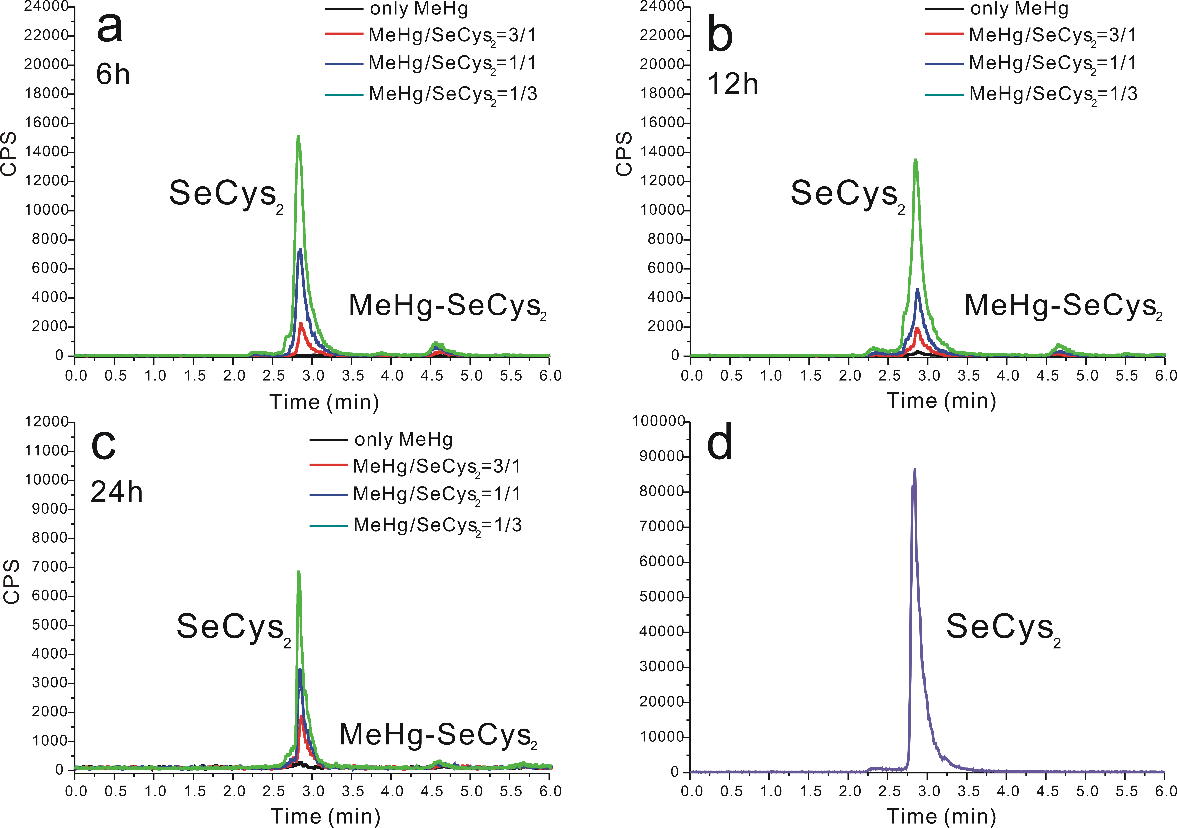


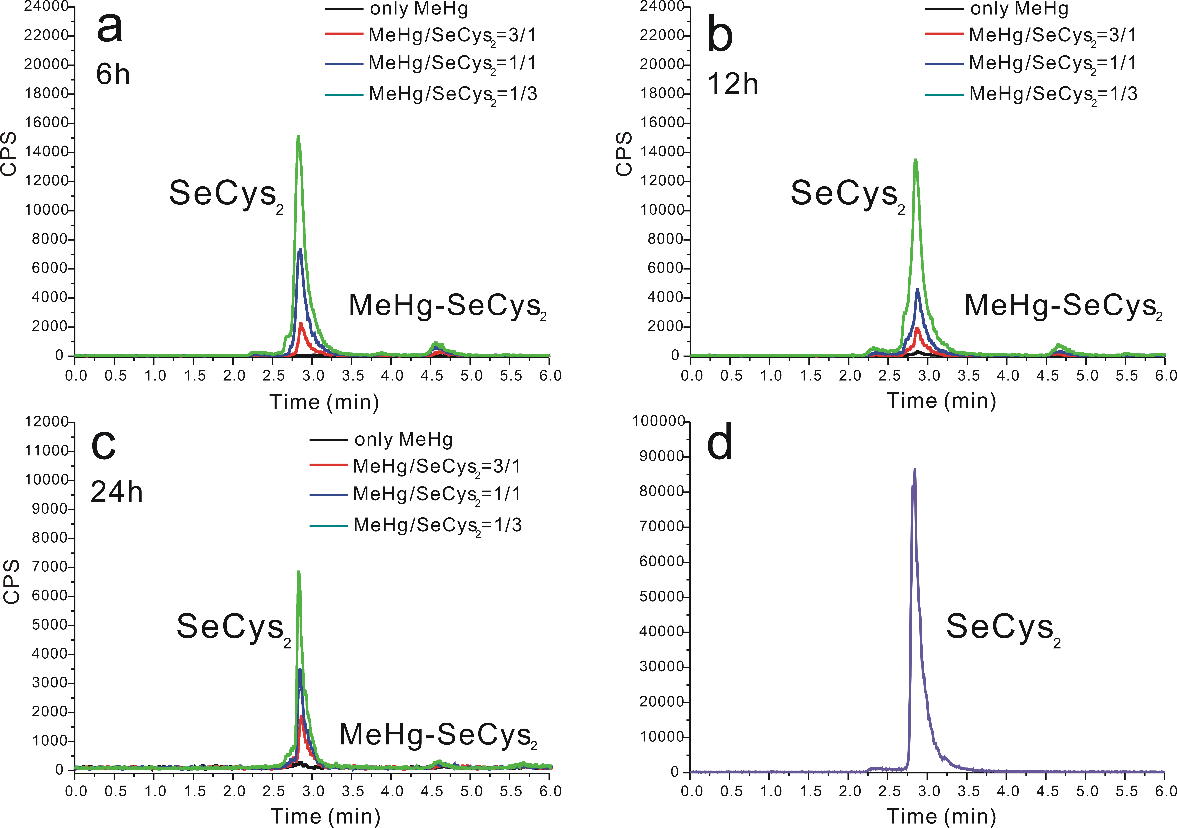


**Figure S11** Chromatograms of selenium species obtained by RP-HPLC-ICP-MS for the culture medium with the cells incubated with MeHg, and co-incubation with MeHg and SeCys_2_ at the ratios of 3:1, 1:1 and 1:3 for 6 h (a), 12 h (b) and 24 h (c), respectively.

**Table S1 Analytical performance for target analytes by ETV-ICP-MS**

|  | LOD  (µg L^-1^) | Linear range  (µg L^-1^) | Linear equation  (µg L^-1^) | R^2^ | RSD^a^  (%) | Recovery  (%) |
| --- | --- | --- | --- | --- | --- | --- |
| MeHg | 0.52 | 2-1000 | y=1407x+552 | 0.9990 | 6.2 | 107±7.2^b^ |
| SeCys_2_ | 0.47 | 2-1000 | y=1819x+858 | 0.9988 | 4.2 | 96±4.3^c^ |

a: n=7, C_MeHg, SeCys2_= 5.0 µg L^-1^; b: spiking 15 µg L^-1^ MeHg in cell suspension; c: spiking 20 µg L^-1^ SeCys_2_ in cell suspension.

**Table S2 Analytical results (means±s.d., n=3) for the determination of mercury complexes in cells cytosol by RP-HPLC-ICP-MS**

| Incubation time | Incubation species | MeHg-GSH  (fg cell^-1^) | MeHg-Cys  (fg cell^-1^) |
| --- | --- | --- | --- |
| 6 h | only MeHg | NQ | 62.3±2.4 |
|  | MeHg/SeCys_2_=3/1 | 89.4±3.5 | 203±8.7 |
|  | MeHg/SeCys_2_=1/1 | 133±7.4 | 256±12 |
|  | MeHg/SeCys_2_=1/3 | 234±7.2 | 297±10 |
| 12 h | only MeHg | NQ | 113±5 |
|  | MeHg/SeCys_2_=3/1 | NQ | 313±12 |
|  | MeHg/SeCys_2_=1/1 | 118±9.3 | 340±14 |
|  | MeHg/SeCys_2_=1/3 | 210±12 | 345±14 |
| 24 h | only MeHg | NQ | 119±5 |
|  | MeHg/SeCys_2_=3/1 | NQ | 330±13 |
|  | MeHg/SeCys_2_=1/1 | NQ | 387±15 |
|  | MeHg/SeCys_2_=1/3 | 182±8 | 423±17 |

NQ: Not quantitation.

**Table S3 Energy summary with respect to MeHg and SeCys_2_**

| Types | Species | ΔE(Kcal mol^-1^) | ΔE_0_(Kcal mol^-1^) | ΔG_298.15_ (Kcal mol^-1^) |
| --- | --- | --- | --- | --- |
| COO…Hg | 1-a | -46.0 | -44.8 | -32.3 |
|  | 1-b | -44.7 | -43.2 | -32.9 |
| Se…Hg | 2-a | -34.9 | -34.0 | -21.7 |
|  | 2-b | -34.8 | -33.8 | -21.6 |
| NH_2_…Hg | 3-a | -48.0 | -45.9 | -32.2 |

Note: The structure of species was referred in Figure S10.

**Table S4** Analytical results (means±s.d., n=3) for the determination of dissociated mercury species in cells cytosol by chip-based on-line MSPME-microHPLC-ICP-MS

| Incubation time | Incubation species | Hg^2+^ (fg cell^-1^) | MeHg^+^ (fg cell^-1^) |
| --- | --- | --- | --- |
| 6 h | only MeHg | 1.6±0.2 | 5.4±0.3 |
|  | MeHg/SeCys_2_=3/1 | 2.2±0.2 | 13.3±0.6 |
|  | MeHg/SeCys_2_=1/1 | 1.8±0.2 | 14.2±1.1 |
|  | MeHg/SeCys_2_=1/3 | 1.6±0.2 | 18.7±1.2 |
| 12 h | only MeHg | 1.8±0.2 | 11.4±0.4 |
|  | MeHg/SeCys_2_=3/1 | 2.0±0.2 | 23.1±1.0 |
|  | MeHg/SeCys_2_=1/1 | 2.5±0.2 | 28.9±2.1 |
|  | MeHg/SeCys_2_=1/3 | 2.2±0.2 | 30.9±1.2 |
| 24 h | only MeHg | 2.9±0.2 | 26.6±0.2 |
|  | MeHg/SeCys_2_=3/1 | 6.5±0.3 | 46.9±2.7 |
|  | MeHg/SeCys_2_=1/1 | 6.3±0.2 | 52.5±0.9 |
|  | MeHg/SeCys_2_=1/3 | 5.9±0.2 | 63.1±0.7 |

**Table S5** Analytical results (means±s.d., n=3) for the determination of mercury species in culture medium by RP-HPLC-ICP-MS

| Incubation time | Incubation species | MeHg-GSH  (µg mL^-1^) | MeHg-Cys  (µg mL^-1^) | MeHg and SeCys_2_ aggregation  (µg mL^-1^) |
| --- | --- | --- | --- | --- |
| 6 h | only MeHg | ND | 0.35±0.02 | ND |
|  | MeHg/SeCys_2_=3/1 | ND | 0.24±0.02 | 0.19±0.01 |
|  | MeHg/SeCys_2_=1/1 | 0.06±0.01 | 0.20±0.01 | 0.33±0.01 |
|  | MeHg/SeCys_2_=1/3 | 0.06±0.01 | 0.18±0.01 | 0.41±0.01 |
| 12 h | only MeHg | 0.19±0.01 | 0.38±0.04 | ND |
|  | MeHg/SeCys_2_=3/1 | 0.24±0.02 | 0.37±0.03 | ND |
|  | MeHg/SeCys_2_=1/1 | 0.27±0.02 | 0.35±0.03 | 0.23±0.01 |
|  | MeHg/SeCys_2_=1/3 | 0.36±0.03 | 0.34±0.01 | 0.46±0.03 |
| 24 h | only MeHg | 0.27±0.03 | 0.40±0.01 | ND |
|  | MeHg/SeCys_2_=3/1 | 0.34±0.02 | 0.38±0.04 | ND |
|  | MeHg/SeCys_2_=1/1 | 0.38±0.05 | 0.37±0.02 | ND |
|  | MeHg/SeCys_2_=1/3 | 0.43±0.02 | 0.34±0.04 | ND |

ND: Not detected.

**Table S6** Optimized operating conditions for online HPLC-ICP-MS

| Operation condition of HPLC |  |
| --- | --- |
| Analytical column | SEC column (Superdex 200 10/300 GL) |
| Mobile phase | 20 mmol L^-1^ NH_4_Ac, pH 7.0 |
| Temperature | 25 ºC |
| Flow rate | 0.4 mL min^-1^ |
| Inject volume | 10 µL |
| Analytical column | RP-C18 column (Elitehplc, Hypersil ODS2, 5 µm, 4.6×250 mm) |
| Mobile phase | 2 mmol L^-1^ methanesulfonic acid, pH 5.0, 6% MeOH |
| Temperature | 25 ºC |
| Flow rate | 1.0 mL min^-1^ |
| Inject volume | 30 µL |
| Analytical column | Micro RP-C18 column (Acclaim, 3 µm, 300Å, 1.0 mm i.d.) |
| Mobile phase | A: 0.1 % mercaptoethanol, 30 mmol L^-1^ NH_4_Ac, pH 7.0; B: methanol |
| Temperature | 25 ºC |
| Flow rate | 40 µL min^-1^ |
| Inject volume | 10 µL |
| Gradient program | 1-8 min, 2% methanol; 8-10 min, 2% to 55% methanol; 10-12 min, 55% methanol; 12-14 min, 55% to 2% methanol; 14-20 min, 2% methanol. |
| Operation condition of ICP-MS |  |
| Rf power | 1350 W |
| Plasma gas flow rate | 15 L min^-1^ |
| Auxiliary gas flow rate | 0.1 L min^-1^ |
| Carrier gas flow rate | 0.9 L min^-1^ |
| Sampling depth | 7.0 mm |
| Scanning mode | Time-resolved analysis |
| Dwell time | 0.1 s |
| Monitored isotope | ^77^Se, ^82^Se,^198^Hg, ^200^Hg, ^202^Hg |

**Table S7** Optimized operating conditions for ETV-ICP-MS

| Temperature programs of graphite furnace ETV-ICP-MS |  |
| --- | --- |
| Modifier | 50 mg L^-1^ Pd(NO_3_)_2_ |
| Drying step | 100 ºC, ramp 5 s, hold 10 s |
| Dyrolysis step | 150 ºC, ramp 5 s, hold 15 s |
| Vaporization step | 2600 ºC, 4 s |
| Cooling step | 100 ºC, 5 s |
| Cleaning step | 2600 ºC, 3 s |
| Operation condition of ICP-MS |  |
| Rf power | 1250 W |
| Plasma gas flow rate | 15 L min^-1^ |
| Carrier gas flow rate | 0.7 L min^-1^ |
| Sampling depth | 7.0 mm |
| Scanning mode | Time-resolved analysis |
| Dwell time | 0.1 s |
| Monitored isotope | ^77^Se, ^82^Se, ^198^Hg, ^200^Hg, ^202^Hg |

Reference

1 Khan, M. A. K., Asaduzzaman, A. M., Schreckenbach, G. & Wang, F. Y. Synthesis, characterization and structures of methylmercury complexes with selenoamino acids. *Dalton Trans.*, 5766-5772 (2009).

2 Falcone, G. *et al.* Sequestering ability of some chelating agents towards methylmercury(II). *Anal. Bioanal. Chem.* **405**, 881-893 (2013).

3 Becke, A. D. A multicenter numerical integration scheme for polyatomic molecules. *The Journal of Chemical Physics* **88**, 2547-2553 (1988).

4 Lee, C., Yang, W. & Parr, R. G. Development of the Colle-Salvetti correlation-energy formula into a functional of the electron density. *Physical Review B* **37**, 785-789 (1988).

5 Delley, B. An all‐electron numerical method for solving the local density functional for polyatomic molecules. *The Journal of Chemical Physics* **92**, 508-517 (1990).

6 Delley, B. The conductor-like screening model for polymers and surfaces. *Molecular Simulation* **32**, 117-123 (2006).
